# Mobile Applications for Learning Hand Hygiene: A Comparative Analysis

**DOI:** 10.3390/healthcare12161554

**Published:** 2024-08-06

**Authors:** Dominika Muršec, Adrijana Svenšek, Lucija Gosak, Sonja Šostar Turk, Urška Rozman, Gregor Štiglic, Mateja Lorber

**Affiliations:** 1 Faculty of Health Sciences, University of Maribor, Žitna Ulica 15, 2000 Maribor, Slovenia; adrijana.svensek1@um.si (A.S.); lucija.gosak2@um.si (L.G.); sonja.sostar@um.si (S.Š.T.); urska.rozman@um.si (U.R.); gregor.stiglic@um.si (G.Š.); mateja.lorber@um.si (M.L.); 2 Faculty of Electrical Engineering and Computer Science, University of Maribor, Koroška cesta 46, 2000 Maribor, Slovenia; 3 Usher Institute, University of Edinburgh, Edinburgh EH8 9YL, UK

**Keywords:** hand hygiene, innovative education, WHO

## Abstract

Infection control is crucial for high-quality patient care. One of the most effective and commonly used infection control procedures is hand hygiene which, it is known, requires repeated refresher training. There are many ways to educate healthcare professionals about hand hygiene, including the use of mobile applications (apps). Our aim is to review such hand hygiene apps, and to identify which have been available since 2021 and to assess their quality. We conducted a review using the PRISMA diagram to document our app selection process in the Google Play Store and Apple store in March 2024. For the evaluation of apps, we used the user version of the Mobile Application Rating Scale questionnaire (uMARS). Of 16 apps only five adhere to WHO hand hygiene guidelines. Timers were included in 12 of the 16 apps and reminders were included in 10 of 16 apps. The highest overall uMARS scoring app was Give Me 5–Hand Hygiene (4.31 ± 0.28), while Wash your hands! (1.17 ± 0.14) had the lowest score. We found that more than half of the apps were unavailable from the 2021 review. We believe that app-based education could effectively sustain hand hygiene knowledge in healthcare settings.

## 1. Introduction

Proper hand hygiene is one of the most important interventions to reduce healthcare-associated infections (HAI) [1,2]. HAIs have consequences not only for patients, but also economically and socially [3], which is why it is particularly important to educate healthcare staff about hand hygiene, as contaminated hands of healthcare workers are the most frequent cause of the transmission of infections [2]. When it comes to hand hygiene, we usually focus on handwashing and hand disinfection. Handwashing with soap and water is recommended for removing visible dirt when hands are greasy and very soiled, while disinfection is recommended especially in the hospital setting as it is effective in reducing the microbial load (e.g., when in direct contact with the patient, before fitting and after removing gloves, and after contact with the patient’s surroundings) [4]. The World Health Organization (WHO) has developed recommendations in the form of the Five Moments for Hand Hygiene to facilitate the training and understanding of health workers on the importance of hand hygiene [5,6]. Reasons for lower hand hygiene compliance among healthcare workers include high workloads [7,8], job burnout [9], motivational factors, perception of the working environment [10], crowding [11], limited resources, and staff shortages [12]. Hand hygiene can be improved through the education of healthcare staff, monitoring, and feedback on performance [13]. Education about hand hygiene is crucial for the delivery of high quality healthcare services, as is the assessment and ongoing monitoring of knowledge [14]. Research shows that hand hygiene compliance is much higher when audiovisual, digital, and innovative learning methods are integrated with traditional teaching methods [15]. One of the possible forms of learning is through apps. Apps already play an important role in medical education as they enable easy access to information and training, and are widely available [16]. Apps facilitate access to information, improve skills, and enable repetition outside the classroom or learning environment [17]. They are useful because they influence changes in behavior and the achievement of goals, e.g., lifestyle- or sustainability-related goals [18]. In the context of hand hygiene, apps contribute to sustainable practices by streamlining audits, providing cost-effective solutions, and offering customizable data points. These apps increase efficiency, accuracy, and actionable insights for healthcare organizations. Their rapid implementation makes them valuable tools for the improvement of infection prevention and control [19,20]. Apps are otherwise used to teach nursing skills [21], basic child life-support [22], improve self-care for patients with type 2 diabetes mellitus [23], for clinical nursing education [24], for health care-associated infection prevention [25], and to support hand hygiene practice [26]. Gasteiger et al. [26] have already reviewed apps involving hand hygiene and assessed their quality, content, functional, and technical features. In their research, they suggested that, in the future, apps should be developed in collaboration with experts and best practice. For this reason, we also decided to review apps on the topic of hand hygiene, with an emphasis on those created after 2021 and to determine their quality. 

## 2. Materials and Methods

We conducted a review of available apps in the Google Play Store [27] and App Store [28] in March 2024. We reviewed apps on mobile phones and tablets. We used the PRISMA (Preferred Reporting Item for Systematic Reviews and Meta-Analyses) [29] flow diagram and PRISMA checklist Supplementary Material File S1 (Table S1)) to improve the transparency and organization of our review, although PRISMA is usually recommended for systematic reviews. Our review of apps was based on the PIO method [30]: the target population (P) consisted of the healthy adult population, the intervention (I) was the identification of smartphone apps for learning proper hand hygiene, and the outcome (O) was the evaluation of the apps in terms of the features included and an assessment of the quality of the mobile apps based on uMARS. This section describes how we collected, selected, and evaluated apps to help us learn about hand hygiene.

### 2.1. Search Strategy

We searched for apps using the keywords “hand hygiene” and “hand washing”. We included apps related to hand hygiene (washing and/or disinfecting) that are freely available (free of charge) and in English. We included apps that are intended for adults. Exclusion criteria included apps in other foreign languages, paid apps, apps without educational content, and apps that were not technically or contextually relevant. We looked at apps in English as these are the most accessible to the widest range of users. We also only included free apps, as these are available to all users in health care services and educational institutions.

### 2.2. Screening

Initially, the first author counted apps on individual devices and mobile stores. After removing duplicates, the first author reviewed them by title and icon, and screened them by description. Then three authors downloaded the apps and assessed them for eligibility. We installed all the included apps on a Samsung Galaxy Tab S6, Samsung Galaxy A13, iPhone 13 pro max, or iPad Pro (3rd generation). Apps that met the inclusion criteria were thus included in the review and their characteristics transferred to Microsoft Excel Version 2407 (Build 17830.20138). The analysis of the credibility of the apps involved three authors. The assessment was carried out on an individual and independent basis. In case of inconsistency, we resolved issues through a discussion between the involved researchers.

### 2.3. Assessing the Overall Quality of Mobile Apps

Three registered nurses, experienced in implementing infection prevention measures including hand hygiene, used all the apps and then assessed the apps based on the uMARS (Mobile Application Rating Scale (user version)) questionnaire [31]. The authors of the uMARS recommend that the app is thoroughly tested and used for at least 10 minutes before evaluation, which was also considered when evaluating the apps. The authors had previously done similar evaluations [22,23]. As we plan to build on the study to assess the long-term evaluations, we decided to use the uMARS, which includes engagement, functionality, aesthetics, and information mean score. Section A (engagement) contains entertainment, interest, customization, interactivity, and target group. Section B (functionality) includes performance, ease of use, navigation, and gestural design. Section C (aesthetics) assesses layout, graphics, and visual appeal. Section D (information) includes quality of information, quantity of information, visual information, and credibility of source. The average of four constructs (Sections A–D) provides the final uMARS quality score. Section E contains app subjective quality, while section F contains the perceived impact items. A five-point Likert scale was used to rate the items in the questionnaire. We also calculated the intraclass correlation coefficient (ICC2k; intraclass correlation coefficient, two-way random, average measures, and absolute agreement) [32,33]. To understand the user experience better, we further analysed user feedback and reviews from Google Play and the App Store. We used a qualitative methodology and looked at the most common themes and issues raised by users in their comments, assessed the frequency of positive and negative comments, and analysed specific feedback highlighting practical issues, usability, and performance of the apps.

### 2.4. Statistical Analysis

We examined continuous variables, considering their Gaussian distribution. The results were reported as means along with standard deviations (SD). The data underwent analysis using the SPSS statistical package version 29.0.0, developed by IBM Inc., New York, NY, USA.

## 3. Results

**Figure 1:**
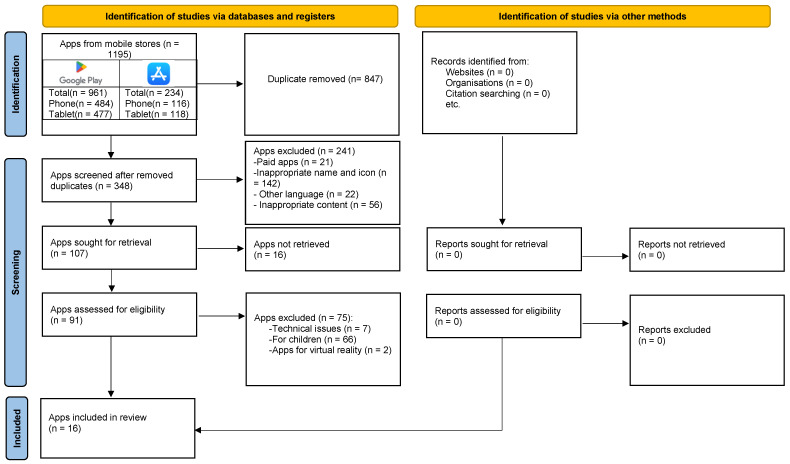
PRISMA flow diagram of the selection of included apps [29].

Using keywords, we found a total of 1195 apps in both mobile stores. We removed 847 duplicates, leaving us with 348 apps. We checked all of them by name and icon. At this point, we eliminated a further 241 apps. Of these, there were 21 paid apps, 142 inappropriate ones in terms of name or icon, 22 in other languages, and 56 with inappropriate content. This left us with 107 apps, which we sought for retrieval. We did not retrieve 16 apps, so we assessed 91 apps for eligibility. At this point, we excluded another 75 apps due to technical issues (*n* = 7) because they were aimed at children (*n* = 66) or because they were for use with virtual reality glasses (*n* = 2). Following the reviewer’s comment, we reviewed the applications again. At this stage we eliminated one application as it was no longer available (*n* = 1). In total, we evaluated 16 apps using the uMARS questionnaire (Figure 1).

All included apps were free. One of the included apps was only available on Google Play (*Hand Washing Reminder*), while 12 were only available on the App Store. Three apps (*SureWash Hand Hygiene, Give Me 5–Hand Hygiene, Bubble Beats Trainer*) were available on both Google Play and the App Store. Five of the sixteen apps (31%) were supported by WHO hand hygiene guidelines (*SureWash Hand Hygiene; Give Me 5–Hand Hygiene; Safe Hands; Semmelweis; Hand Washing Reminder*). One of the remaining apps (*Wash your Hands!*) directed users to the CDC (Centers for Disease Control and Prevention) website, where they could read more about proper hand hygiene, but it was not possible to see the right-hand hygiene procedure within the app itself. Two of the sixteen (12.5%) apps included gamification (*Give Me 5–Hand Hygiene and Bubble Beats Trainer*) in the forms of audible feedback, levels, and a point system. Twelve of the sixteen apps (75%) in the final evaluation included a timer, while ten of the sixteen apps (63%) included a reminder. Four apps offered feedback to users, including *Bubble Beats Trainer*, where users collect points in the game, and *Smart HandWash*, where users must open a real faucet and wash their hands, which is detected by a microphone. Five apps (31%) included a video with an actual demonstration of proper hand hygiene, while the rest contained only pictures or none. We sorted the applications by category, namely three (19%) in the education category, ten (63%) in the health and fitness category, two (13%) in utilities, and one (6%) in the lifestyle category. More basic characteristics of the applications are given in the Supplementary Material File S2 (Table S2).

The average quality rating based on the uMARS scale was 2.61 (±0.26). The apps with the highest overall uMARS app quality score were *Give Me 5–Hand Hygiene* (4.31 ± 0.30), followed by *SureWash Hand Hygiene* (4.06 ± 0.35) and *Bubble Beats Trainer* (3.57 ± 0.22). The app *Wash our Hands!* (1.17 ± 0.14) had the lowest mean uMARS app quality score. In the category rating engagement, the apps achieved the following uMARS scores: *Give Me 5–Hand Hygiene* (4.27 ± 0.42), followed by *Smart HandWash* (3.87 ± 0.12), and the lowest mean was in app *Wash your Hands*! (1.07 ± 0.12). In category functionality, the following scores were achieved: *Give Me 5–Hand Hygiene* (4.92 ± 0.14), followed by *SureWash Hand Hygiene* (4.42 ± 0.14). The lowest mean score was recorded for *Wash your Hands!* (1.25 ± 0.25). In the aesthetics category, the following uMARS scores were recorded: *SureWash Hand Hygiene* (4.44 ± 0.38), followed by *Give Me 5–Hand Hygiene* (4.33 ± 0.58); the lowest average uMARS score was recorded for *Wash your Hands!* (1.00 ± 0.00). *SureWash Hand Hygiene* (5.00 ± 0.00) achieved the highest, while *Washo* and *Clean Hands* (1.00 ± 0.00) achieved the lowest scores in the information category. The app *Give Me 5–Hand Hygiene* had the highest mean score in the app subjective quality category (4.39 ± 0.24), followed by *SureWash Hand Hygiene* (3.61 ± 0.32); the lowest mean score for app subjective quality (1.00 ± 0.00) was achieved by *Wash your Hands!* The highest mean score in app perceived impact (4.11 ± 0.59) was achieved by *Give Me 5–Hand Hygiene*. Apps *Wash your Hands!*, *HowToWashYourHands*, and *Clean Hands* achieved the lowest mean scores in the category of perceived impact (Table 1).

The category with the highest overall uMARS score was functionality (3.53 ± 0.20), and the category with the lowest overall uMARS score was app subjective quality (2.14 ± 0.24).

For the overall uMARS score, we calculated inter-rater reliability using ICC, which showed excellent reliability (ICC2, k: 0.96, 95% CI 0.95–0.96).

We analysed specific feedback highlighting practical problems, usability, and effectiveness of the apps. Only rarely did we find that users gave their opinions in words. Of 16 apps, only 3 had comments in the review.

For the *Wash Your Hands!* app, there were six comments from 2020. Of the six positive comments, one was negative, namely “The app freezes on startup”. Common themes and issues that emerged in the comments were: Reminders: several users praise the app for effective handwashing reminders; Usability: users appreciate the app’s help with regular handwashing, which is especially important during the COVID-19 pandemic; Technical issues: reports of the app freezing on startup, suggestions for improving the timer, and adding reminders for cleaning other surfaces.

The *Bubble Beats Trainer* app had six positive comments posted in 2023 and 2024. Common themes and perceived issues were: Educational features: several users pointed out that the app effectively teaches proper hand washing techniques; Fun games: users appreciated that the app included educational content through fun games, which is particularly appealing to children; UV light: the UV light feature, which showed unwashed microbes, was highly appreciated; Ease of use: users pointed out that the app was easy to use and suitable for a wide audience; Customisation: the ability to customise the app, such as adding your own articles, was appreciated.

The *LatherApp Hand Wash Timer* app had five positive comments and one negative one from 2020 to 2024, based on: “App doesn’t work”. Common themes and issues: Handwashing Tracking: most users appreciated the feature that helped them keep track of their handwashing schedule; Apple Watch Compatibility: users were satisfied with the app’s performance on the Apple Watch Series 3, which is commendable; Use of Timers: the app allowed you to set timers for different activities throughout the day.

Listening to Music: the ability to listen to your favourite music while washing your hands was appreciated; Technical Issues: one user reported serious technical problems where the app did not work properly and they marked it unusable; Wish for Additional Features: some users would like to have seen additional features, such as the ability to view handwashing history and more information about song settings.

## 4. Discussion

We found a variety of apps for teaching hand hygiene. More than half of the apps included a timer. A quarter of the apps were supported by WHO hand hygiene guidelines and included videos of real hand washing. Less than a quarter of the apps included user feedback and gamification. None of the apps included AI-based interactions or educational material. The quality of the apps varied widely. The highest scores were in the functional category, and the lowest in the app subjective quality category. The app with the highest overall uMARS score was Give Me 5–Hand Hygiene. This app was the best because it was interesting, graphically appealing, interactive, contained elements of gamification, offered feedback, was supported by WHO guidelines, contained video material, and allowed the testing of knowledge in the form of a quiz. Wash your hands! was the lowest-rated mobile app. With this app, there was only the option to set a reminder and a timer. No proper hand hygiene procedure was presented, only text urging users to read the United States Centers for Disease Control and Prevention recommendations.

With the technology market expanding and apps being developed rapidly, we looked at apps that teach hand hygiene. Gasteiger et al. [26] had already reviewed apps for hand hygiene education, and we wanted to know how many of these apps were still available and how many new ones had been developed in the meantime. Therefore, we decided to review and compare apps for learning hand hygiene. During our review, we came across two potentially relevant apps, but they required virtual reality headsets to run, so we eliminated them. We also excluded some applications for hand hygiene observation that required registration with the institution to function. Compared with the previous article, the three apps we included in the final evaluation were no longer available in Google Play [27]. Seventy (70) apps evaluated in the previous review [26] were no longer available in mobile stores. Three apps we found were only available for older versions of mobile phones or tablets, so we did not include them in the review. Many articles so far have focused on apps related to hand hygiene monitoring and observation [34,35,36], which we did not include, as they mainly required a login with an institutional account and did not show the correct hand hygiene procedure. Baggio et al. [37] also reviewed apps, assessed their quality in hand hygiene education, and determined their utility for healthcare professionals. They concluded that apps can be a good complement to existing educational methods. We also found other research that did not only focus on hand hygiene when reviewing apps. In the review and analysis of apps for the prevention of infections related to healthcare on mobile phones, Schnall and Iribarren [25] also included two apps related to hand hygiene. Gu et al. [38] determined the effectiveness of a game-based app in teaching about flushing and locking venous catheters with saline-filled syringes among nursing students. In part of the research, they found that the incidence of hand hygiene errors in the app group was lower than in the control group. We found that most of the apps included were developed during the COVID-19 pandemic or later, as evidenced by other studies [39,40]. The only applications that achieved a quality above four belonged to the education category. Only three applications were also descriptively evaluated in mobile stores. The *Wash Your Hands app!* received several positive comments and one negative one, and it received the lowest score among the evaluated applications in the uMARS questionnaire. The Bubble Beats Trainer app came third among our rated apps, while online store users raved about it. The *LatherApp Hand Wash Timer* app also received a few positive comments and one negative comment.

The study by Gonzalez et al. [41] found that e-learning is just as effective and much more engaging than traditional teaching methods. We agree that it is essential to integrate education through different technologies in the training of students and health professionals: apps, virtual reality, e-learning, etc. Research by Sung et al. [42] has also shown that the impact of using mobile phones as tools in education is better than using desktop computers or other devices. One study found that audio–visual media such as mobile phones, practical simulations, and videos improved hand hygiene knowledge and skills among healthcare workers [15]. The rapid growth nowadays of the use of artificial intelligence in various fields of education also encompasses the learning of hand hygiene [43,44]. Nagar et al. [44] highlighted the use of neural networks in hand hygiene monitoring and compliance systems as an important factor in containing the spread of infections. Similarly, Fitzpatrick et al. [45] pointed out that artificial intelligence could play an important role in detecting transmission events and present opportunities for improving diagnostics.

The main limitations of our article are that only three authors evaluated mobile applications, that we excluded paid applications, that the market and developers of apps change rapidly, and that, despite following the PRISMA diagram, we did not use a checklist, as it was not a typical systematic review. It is also a limitation that the paper focused only on reviewing and assessing the quality of the apps, and did not evaluate the user experience or the effectiveness of the app on hand hygiene. Despite these limitations, we believe that our study provides important insights into current trends and the quality of hand hygiene apps, which can serve as a basis for further research. For future research, we suggest that mobile applications be tested, e.g., on students, and we can then find out how effective they are in terms of education and sustainability. In future, we intend to include long-term studies to assess the sustainability and effectiveness of these apps (this includes monitoring the use of the apps over a longer period of time (e.g., 6 months, 1 year), measuring changes in hand hygiene knowledge and practice among users, and analysing the maintenance and upgrading of the apps by the developers). It would also make sense to develop an application supported by elements of artificial intelligence.

## 5. Conclusions

Apps can be a good support for standard educational methods, including hand hygiene. Through a review of mobile apps for hand hygiene education in two mobile stores, we found that their quality varied. There is a need for apps to have the support of WHO guidelines or other relevant institutions and, because of that, it is necessary that professionals are included in the development of apps. For the future, it is important that mobile apps are developed according to current guidelines and that they are critically evaluated with valid questionnaires or tools, as this is the only way to ensure quality and usable mobile apps for hand hygiene. It would also make sense for a larger number of end users to provide the rating. We also propose a review of existing solutions for learning hand hygiene using artificial intelligence, which is also developing strongly in this field.

## Figures and Tables

**Table 1:** uMARS means scores.

Name	Engagement (SD ^1^)	Functionality (SD)	Aesthetics (SD)	Information (SD)	App Quality (SD)	App Subjective Quality (SD)	App Perceived Impact (SD)
SureWash Hand Hygiene	3.40 (±0.20)	4.42 (±0.14)	4.44 (±0.38)	5.00 (±0.00)	4.06 (±0.35)	3.61 (±0.32)	3.61 (±0.19)
Give Me 5–Hand Hygiene	4.27 (±0.42)	4.92 (±0.14)	4.33 (±0.58)	4.92 (±0.14)	4.31 (±0.28)	4.39 (±0.24)	4.11 (±0.59)
Ultra Wash	2.33 (±0.50)	3.67 (±0.29)	2.67 (±0.58)	1.42 (±0.29)	2.12 (±0.73)	1.75 (±0.25)	2.17 (±1.26)
Wash your Hands!	1.07 (±0.12)	1.25 (±0.25)	1.00 (±0.00)	1.58 (±0.14)	1.17 (±0.14)	1.00 (±0.00)	1.83 (±1.44)
Wash hands	2.40 (±0.40)	3.67 (±0.29)	2.67 (±0.58)	1.42 (±0.29)	2.14 (±0.73)	1.75 (±0.25)	2.17 (±1.26)
Semmelweis	2.80 (±0.00)	3.83 (±0.14)	3.22 (±0.51)	3.75 (±0.66)	3.05 (±0.92)	2.33 (±0.14)	2.89 (±0.54)
Safe Hands	3.40 (±0.20)	3.75 (±0.25)	3.67 (±0.33)	4.00 (±0.43)	3.41 (±0.66)	2.56 (±0.27)	2.89 (±0.54)
HowToWashYourHands	1.73 (±0.12)	3.50 (±0.25)	1.56 (±0.19)	1.33 (±0.29)	1.92 (±0.23)	1.25 (±0.25)	1.83 (±1.44)
Smart HandWash	3.87 (±0.12)	3.08 (±0.14)	2.78 (±0.19)	1.58 (±0.29)	2.78 (±0.11)	2.78 (±0.05)	3.00 (±0.44)
Washo	1.60 (±0.00)	3.33 (±0.14)	1.56 (±0.19)	1.00 (±0.00)	1.78 (±0.11)	1.17 (±0.29)	1.83 (±1.44)
Bubble Beats Trainer	3.67 (±0.23)	3.92 (±0.14)	4.22 (±0.69)	3.08 (±0.29)	3.57 (±0.22)	3.42 (±0.38)	3.22 (±0.25)
LatherApp Hand Wash Timer	1.60 (±0.00)	3.42 (±0.14)	1.56 (±0.19)	2.17 (±0.29)	2.01 (±0.31)	1.36 (±0.38)	2.00 (±1.30)
Wash Your Hands Streaks	1.60 (±0.00)	3.42 (±0.14)	1.56 (±0.19)	1.67 (±0.29)	1.93 (±0.24)	1.36 (±0.38)	2.00 (±1.30)
Washy hands timer	2.20 (±0.20)	4.08 (±0.14)	3.11 (±0.19)	2.83 (±0.29)	2.97 (±0.26)	2.53 (±0.21)	2.78 (±0.63)
Clean Hands	1.60 (±0.00)	2.83 (±0.14)	1.56 (±0.19)	1.00 (±0.00)	1.72 (±0.05)	1.17 (±0.29)	1.83 (±1.44)
Hand Washing Reminder	2.27 (±0.23)	3.25 (±0.43)	2.56 (±0.19)	1.33 (±0.14)	2.41 (±0.14)	2.39 (±0.32)	2.61 (±0.77)
Overall mean (SD)	2.48 (±0.19)	3.53 (±0.20)	2.66 (±0.32)	2.32 (±0.24)	2.56 (±0.36)	2.14 (±0.24)	2.52 (±0.95)

^1^ SD—Standard deviation.

## Data Availability

No additional data.

## Supplementary Materials

The following supporting information can be downloaded at: https://www.mdpi.com/article/10.3390/healthcare12161554/s1, Supplementary Material File S1 (Table S1: PRISMA Checklist) and Supplementary Material File S2, Table S2: mobile application characteristics.
