# Supplementary material for: Mobile Applications for Learning Hand Hygiene: A Comparative Analysis
Source: Healthcare (Basel). 2024 Aug 6;12(16):1554. doi: 10.3390/healthcare12161554 (PMC11353288; doi:10.3390/healthcare12161554)
Supplement: Supplementary file 1 [file healthcare-12-01554-s001.zip › Supplementary Material File S2.pdf]

Supplementary Material File S2 "Additional information about included applications"

Table S2. Mobile applications characteristic

Name and icon	Market	Developer, developer's website and country	Category	Version	First published	Updated	Downloads	User rating (nr. of reviewers)
SureWash Hand Hygiene 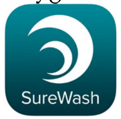	Google Play; Apple	GLANTA Ltd https://surewash.com/ Ireland	Education	Google Play: 2.2.1; Apple: 2.2.1 (iPhone and iPad)	Google Play: Mar 15, 2018; Apple: 6 years ago	Google Play: May 12, 2020 Apple: 2 years ago	Google Play: 1,000+; Apple: N/A	Google Play: 4.2 (12); Apple: No ratings
Give Me 5 – Hand Hygiene 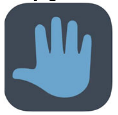	Google Play; Apple;	Forum Service s. r. l. https://www.forumservice.net/; Italy	Education	Google play: 1.2.0; Apple: only on iPad	Google Play: May 4, 2018; Apple: N/A	Google Play; Sep 21, 2018 Apple: N/A	Google Play: 1,000+; Apple: N/A	Google Play: 2.6 (5); Apple: No ratings
Ultra Wash 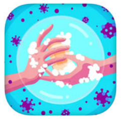	Apple	Dharmeshi Khudaiya http://www.netdroidtech.com/; India	Health & Fitness	All devices: 1.0 or later	N/A	N/A	N/A	No ratings
Wash your Hands! 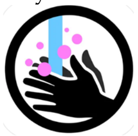	Apple	Round Trip Productions, llc https://www.goroundtrip.co/	Health & Fitness	All devices: 1.0.2	4 years ago	4 years ago	N/A	4.8 (17)
Wash hands 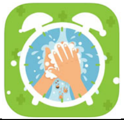	Apple	ECU=SHOP, https://ecu-shop.com/; Thailand	Utilities	1.01 or later (iPhone and iPod touch)	4 years ago	4 years ago	N/A	N/A

Semmelweis	Apple	Leandro Fournier; URL not available, Argentina	Education	All devices: 1.4	4 years ago	3 years ago	N/A	5.0 (4)
Safe Hands	Apple	SectorQube Inc https://sectorqube.com/ India	Lifestyle	All devices: 1.0	N/A	N/A	N/A	N/A
HowToWashYourHands	Apple	Tiziano Pieretti; https://tizianopieretti.wordpress.com/2020/03/03/washyourhands/; Italia	Health & Fitness	Only on iPhone; Verison: N/A	N/A	N/A	N/A	N/A
Smart HandWash	Apple	Innovation Factory Limited https://www.innovfactory.com/ United Kingdom	Health & Fitness	All devices: 1.1	4 years ago	4 years ago	N/A	N/A
Washo	Apple	Pranav Karthik Mruthyunjayan; URL not available	Health & Fitness	All devices: 1.0 or later	N/A	N/A	N/A	N/A
Bubble Beats Trainer	Google Play; Apple	Indelible Learning, Inc. https://indeliblelearning.com/ United States of America	Health & Fitness	Google Play: 3.1.0; Apple: 3.1.0 (all devices)	Google Play: May 9, 2023 Apple: 1 years ago	Google Play: Feb 21, 2024 Apple: 9 months ago	Google Play: 10+ Apple: N/A	Google Play: N/A; Apple: 5.0 (14)
LatherApp Hand Wash Timer	Apple	Think Tap Work https://thinktapwork.com/	Health & Fitness					

		United States of America		All devices: 2.2	4 years ago	2 years ago	N/A	4.1 (29)
Wash Your Hands Streaks	Apple	Reid Fleishman https://reidf.net/ United States of America	Health & Fitness	All devices: 1.2	4 years ago	11 months ago	N/A	5.0 (3)
Washy hands timer	Apple	Panda Play Studios (PTY) LTD https://www.pandaplaystudios.com/ South Africa	Utilities	All devices: 1.0 or later	N/A	N/A	N/A	3.5 (2)
Clean Hands	Apple	Mark Manser; https://xwash.xappsios.com/privacy; N/A	Health & Fitness	All devices: 1.2	4 years ago	1 years ago	N/A	5.0 (3)
Hand Washing Reminder	Google Play	MEFAK	Health & Fitness	1.0.6	Mar 21, 2020	Jun 5, 2023	1,000+	4.5 (31)
